# Deep neural network based artificial intelligence assisted diagnosis of bone scintigraphy for cancer bone metastasis

**DOI:** 10.1038/s41598-020-74135-4

**Published:** 2020-10-12

**Authors:** Zhen Zhao, Yong Pi, Lisha Jiang, Yongzhao Xiang, Jianan Wei, Pei Yang, Wenjie Zhang, Xiao Zhong, Ke Zhou, Yuhao Li, Lin Li, Zhang Yi, Huawei Cai

**Affiliations:** 1 Laboratory of Clinical Nuclear Medicine, Department of Nuclear Medicine, West China Hospital of Sichuan University, No. 37 Guo Xue Alley, Chengdu, 610041 People’s Republic of China; 2 Machine Intelligence Laboratory, College of Computer Science, Sichuan University, Chengdu, 610065 People’s Republic of China

**Keywords:** Metastasis, Cancer, Mathematics and computing

## Abstract

Bone scintigraphy (BS) is one of the most frequently utilized diagnostic techniques in detecting cancer bone metastasis, and it occupies an enormous workload for nuclear medicine physicians. So, we aimed to architecture an automatic image interpreting system to assist physicians for diagnosis. We developed an artificial intelligence (AI) model based on a deep neural network with 12,222 cases of ^99m^Tc-MDP bone scintigraphy and evaluated its diagnostic performance of bone metastasis. This AI model demonstrated considerable diagnostic performance, the areas under the curve (AUC) of receiver operating characteristic (ROC) was 0.988 for breast cancer, 0.955 for prostate cancer, 0.957 for lung cancer, and 0.971 for other cancers. Applying this AI model to a new dataset of 400 BS cases, it represented comparable performance to that of human physicians individually classifying bone metastasis. Further AI-consulted interpretation also improved human diagnostic sensitivity and accuracy. In total, this AI model performed a valuable benefit for nuclear medicine physicians in timely and accurate evaluation of cancer bone metastasis.

## Introduction

Advanced malignant carcinomas, such as breast cancer, prostate cancer, and lung cancer, frequently develop into bone metastasis. Thus the early detection of bone metastasis holds a valuable benefit for choosing the treatment strategy to obtain a better overall survival period and improved life quality of patients^1,2^. Bone scintigraphy (BS) with ^99m^Tc-MDP is one of the most commonly utilized diagnostic techniques to identify bone metastasis in cancer patients since it has merit for whole-body detection and high sensitivity^3^. The latest national survey has reported that more than 1.15 million bone scans were annually performed in China, which occupies a great workload for nuclear physicians. However, the limited resolution of BS images makes the interpretation is time-consuming and experience-dependent work and has the disadvantages of subjectivity, error distinctive, and unsatisfied efficiency.

Recently, the development of artificial intelligence (AI) is creeping into every facet in modern life by its advances in big-data retrieval and explicit feature evaluation, which is ideal for medical image analysis^4–6^. With the help of deep neural networks (DNNs), the computational methods allow an algorithm to program itself by learning from a large set of examples that demonstrate the desired behavior, removing the need to specify rules explicitly^7,8^. Compared to traditional image processing methods, deep learning is more reliable and efficient, since it could automatically extract image features instead of hand-crafted features^9,10^. By now, the AI with deep neural networks have achieved great success in the applications of medical image analysis^11^, such as contouring of nasopharyngeal carcinoma volumes^12^, retinopathy of prematurity screening^13^ and diagnosing of breast ultrasonography images^14^. Considering these advances, the application of DNNs-based AI system in analysis of nuclear BS image is worth pursuing.

In this study, we constructed an AI model based on a DNN with 12,222 cases of ^99m^Tc-MDP bone scintigraphy images from patients with definite clinical conclusions. Then, the diagnostic performance and the consulting potential of AI model for improving human diagnostic accuracy and efficiency were evaluated.

## Methods

### Collection, inclusion, and exclusion of patients

This study with retrospective information collection was approved by the Institutional Ethics Committee of West China Hospital in Sichuan University. We collected 13,477 cases of BS images from patients suspected to have bone metastasis and underwent whole-body BS between January 1st, 2016, and June 30th, 2018. Then, cases with improper injection, improper imaging process, the patients who had definite primary bone tumor, and the ones did not undergo follow-up examinations were excluded.

### Scanning process and diagnostic criteria of cases

Whole-body anterior and posterior views were performed using two gamma cameras (GE Discovery NM/CT 670 and Philips Precedence 16 SPECT/CT). The patient received 555 to 740 MBq of technetium-99 m methylene diphosphonate (^99m^Tc-MDP; purchased from Syncor Pharmaceutical Co., Ltd, Chengdu, China) by intravenous injection, and the images were obtained approximately 3 h post injection. The gamma cameras were equipped with low-energy, high-resolution, parallel-hole collimators. The scan speed was 16–20 cm/min, and the matrix size was 256 × 1024. The energy peak was centered at 140 keV with 15% to 20% windows.

Each BS examination contained two images of anterior and posterior views with resolutions of 256 × 1024. All images collected for data set were DICOM format and interpreted for the presence or absence of bone metastasis via consensus by two nuclear medicine physicians with more than 10 years of experience. The follow-up scans were used to observe whether hot spots had disappeared, remained unchanged, or decreased or increased in size and intensity. The final clinical assessment of bone metastasis was patient-based analysis, it would be determined by clinical assessments which based on the follow-up bone scans and the other radiographic images (Such as SPECT/CT, MRI, and CT et al.)^15^. When no clinical or radiographic signs was found in image, data would be considered Grade 0 with no probability of bone metastasis. If the visible hot spots have confirmed fractures or degenerative changes in gathered clinical records, or the spots disappeared, remained unchanged, or decreased in size and intensity on the follow-up scan and the other radiographic images indicates these lesions leaned away from malignancy and toward the low probability of bone metastasis as Grade 1. However, cases in Grade 2 represents visible hot spots, with localization, distribution, and intensity not typical of degenerative changes or fractures, not substantially changed in scintigraphic follow-up and the radiographic modalities are equivocal, but the overall clinical judgement indicates probable bone metastasis. Grade 3 images had typical scintigraphic or radiographic patterns for bone metastasis and the patient’s medical record states bone metastasis. Thus, Grade 2 and Grade 3 were identified as bone metastasis.

### Cohorts and network architecture for AI model

To obtain an accurate testing results, 12,222 images were randomly assigned to three cohorts: (a) a training cohort of 9776 patients for DNNs construction, (b) a validation cohort of 1223 patients for optimization of the DNNs hyperparameters, and (c) a testing cohort of 1223 patients to test the performance of the model. As shown in Table 1, the images used in our study contained 6021 cases with lung cancer, 1844 cases with prostate cancer, 2100 cases with breast cancer, and 2257 cases with other cancers (37 kinds of cancers were listed in Supplementary Table S1).

**Table 1:** The distribution of training, validation, and testing cohorts for AI modeling.

Characteristic	Lung cancer	Prostate cancer	Breast cancer	Other cancers	Total
**Training cohort**					
Number	4817	1474	1680	1805	9776
Sex					
Male	2699	1474	10	1238	5421
Female	2118	0	1670	567	4355
Age					
Mean + SD	58.5 + 10.8	71.0 + 8.7	51.4 + 10.4	56.1 + 12.9	58.7 + 12.3
< 60 years	2400	137	1296	1054	4887
≥ 60 years	2417	1337	384	751	4889
Skeletal lesions					
No metastasis	2697	756	1066	1138	5657
Metastasis	2120	718	614	667	4119
Metastasis rates	44.01%	48.71%	36.55%	36.95%	42.13%
**Validating cohort**					
Number	602	185	210	226	1223
Sex					
Male	336	185	0	147	667
Female	266	0	210	79	556
Age					
Mean + SD	58.6 + 10.5	71.5 + 8.4	50.3 + 10.3	57.0 + 12.4	58.8 + 12.2
< 60 years	304	15	175	122	616
≥ 60 years	298	170	35	104	607
Skeletal lesions					
No metastasis	337	95	133	142	707
Metastasis	265	90	77	84	516
Metastasis rates	44.02%	48.65%	36.67%	37.17%	42.19%
**Testing cohort**					
Number	602	185	210	226	1223
Sex					
Male	357	185	0	150	692
Female	245	0	210	76	531
Age					
Mean + SD	58.5 + 10.9	71.0 + 8.1	51.5 + 10.0	56.3 + 13.4	58.8 + 12.3
< 60 years	303	14	163	139	619
≥ 60 years	299	171	47	87	604
Skeletal lesions					
No metastasis	337	95	133	142	707
Metastasis	265	90	77	84	516
Metastasis rates	44.02%	48.65%	36.67%	37.17%	42.19%

**Figure 1:**
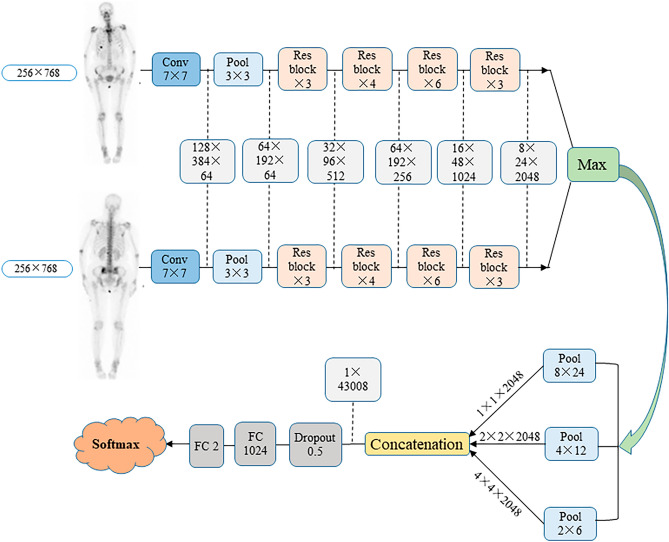
The architecture of convolutional neural network for AI model.

Then, we proposed a multi-input convolutional neural network (CNN) which can accept multiple images as input. The original images (DICOM format) were resized to 256 × 768 and Hu matrix were normalized to [0, 1] before going to the model. Previous studies indicate that fine-tuning with pre-trained networks is an effective method for training CNNs^16,17^. In this study, several ImageNet pretrained networks are explored and ResNet-50 has been chosen to extract high-level features from input images. Fully connection layer was removed from the final layer of ImageNet pretrained network ResNet-50 for feature extraction. The proposed network contains three parts. In the first part, ResNet-50 network was employed to extract high-level features. In the second part, max aggregation operator was used to aggregate high-level features extracted from two images. Since hotspots in the images usually present variant scales. Inspired by spatial pyramid pooling, three pooling layers with different kernel size were used to capture different scale information. In the final part, two fully connected layers were applied to classify the features into metastasis or non-metastasis. The detailed network architecture is shown in Fig. 1.

### Evaluation of AI performance

Performance of the automated AI model was evaluated by the ROC analysis and AUC measurement using the testing cohort containing another 1223 cases. Total cases were divided as 4 subgroups by cancer types: prostate cancer (15.13%), breast cancer (17.17%), lung cancer (49.22%), and other cancers (18.48%), while the sensitivity, specificity, accuracy, PPV, and NPV in each cancer were calculated respectively. Gender and age related diagnostic performance was conducted to investigate whether these factors would affect the results by comparing the AUC values of male versus female, and patient’s age < 60 years versus ≥ 60 years in these patients.

Then, an individual interpreting competition between AI and three nuclear physicians who had more than 5 years’ experience was carried out. A new dataset containing 200 cases with cancer bone metastasis and 200 without metastasis were randomly chosen from 2786 examinations with confirmed conclusion between July and October 2018 in West China Hospital. In this competition, AI and physicians were blinded to the ground truth and distribution of patients, and interpreted images without extra radiologic and medical information, but only based on BS images. To further estimate the potential value of AI model, one hundred days later, these three physicians were required to re-interpreting the same test cohort of 400 cases, and they would give the final judgement after consulting AI’s result. The time–cost, diagnostic sensitivity, specificity, accuracy, PPV, and NPV of AI system and physicians were evaluated, respectively.

### Statistical analysis

In this study, the comparisons of accuracy, sensitivity, specificity, PPV, and NPV between each cancer type were evaluated using the Chi-square test. All analyses were performed by using statistical software SPSS 21.0 (SPSS Inc, Chicago, IL, USA). Statistical significance was considered at the value of *P* < 0.05.

### Ethics approval and consent to participate

This retrospective study was performed in accordance with the Declaration of Helsinki declaration and its later amendments or comparable ethical standards. The ethical permission for the retrospective study was obtained at the Biomedical Research Ethics Committee of West China Hospital of Sichuan University (Approval No. 2019–317), and the requirement to obtain informed consent was waived.

## Results

### Patient characteristics

The flow diagram of case inclusion in this study is shown in Supplementary Table S2. At the beginning, we collected 13,477 images from individual patients who were suspected to have bone metastasis and underwent whole-body BS. Then, 796 patients who had primary bone tumor were excluded, 147 cases were excluded because of poor image quality, and 312 patients were excluded for they did not undergo follow-up examinations. Finally, 12,222 patients (median age, 58.7 ± 12.3 years; male, 6781; female, 5441) were collected and stratified sampling for dataset, including 9776 cases for training, 1223 cases for validating, and another 1223 cases for testing.

### Performance of the AI model

After training and validating process, our AI model indicated considerable diagnostic accuracy of 93.38% in cancer bone metastasis in total of 1223 testing cases, which is better than other models in previous reports (Table 2).

**Table 2:** Comparison of diagnostic performance for cancer bone metastasis by our and previous AI models.

	EB	BN1	BN2	Ours
**Country**	Swedish	Japan	Japan	China
**Training cohort**	795	904	1532	9776
Bone metastases	33%	16%	42%	42%
Age	66 ± 12	64 ± 12	64 ± 12	58 ± 12
Gender				
Male	514	457	790	5421
Female	281	447	742	4355
Cancer types				
Prostate	431	267	451	1474
Breast	217	383	624	1680
Lung	/	/	/	4817
Others	147	254	457	1805
**Validating cohort**	NM	NM	NM	1223
**Testing cohort**	384	257	503	1223

Performance measured by AUC				
**AUC of cancer types**				
Prostate	0.939	0.949	0.957	0.955
Breast	0.847	0.91	0.924	0.988
Lung	–	–	–	0.957
Other	0.77	0.861	0.914	0.971
Total	0.858	0.91	0.932	0.964
**AUC of gender**				
Male	0.877	0.912	0.934	0.963
Female	0.831	0.91	0.932	0.966
**AUC of age**				
< 60 years	–	–	–	0.979
≥ 60 years	–	–	–	0.949

*EB*  EXINIbone^18^, *BN1* BONENAVI version 1^19^, *BN2* BONENAVI version 2^20^.

**Figure 2:**
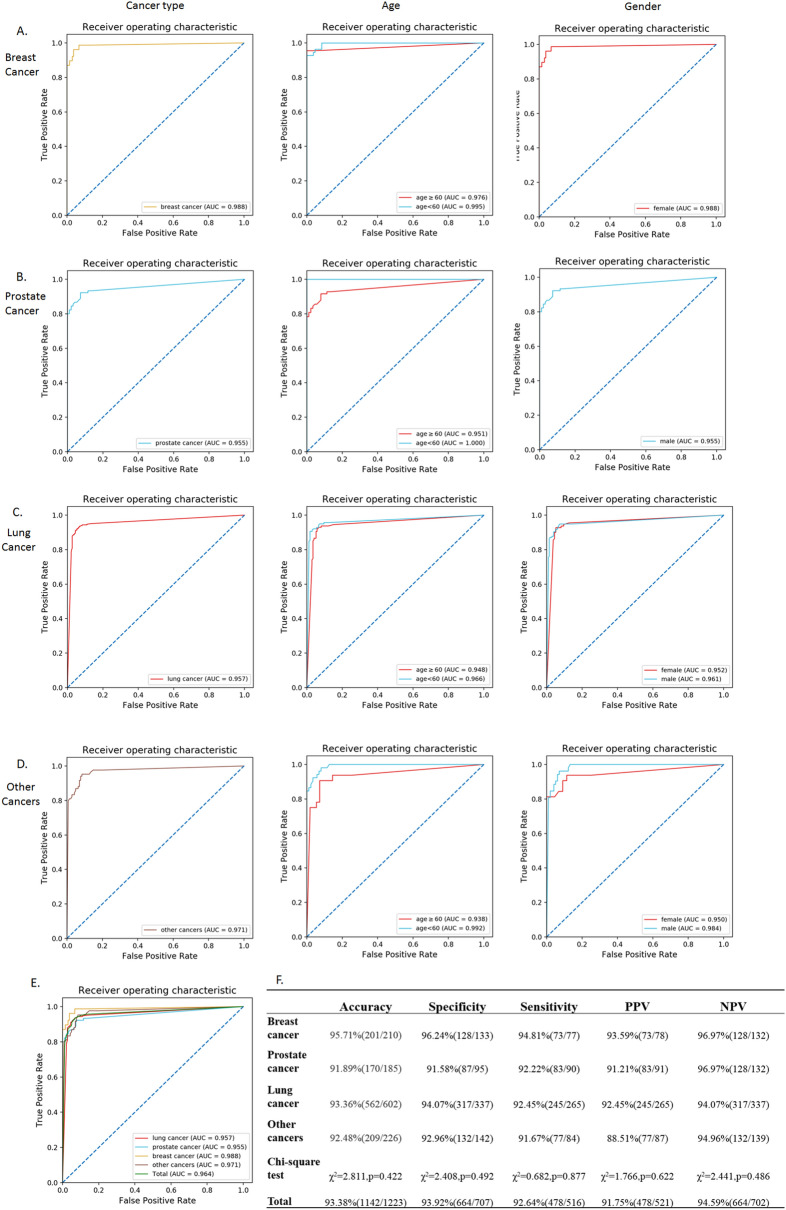
Diagnostic performance of AI model in BS interpretation assessed by ROC analysis for cancer types, age (B) and gender (C) factors. (**A**) Breast cancer; (**B**) prostate cancer; (**C**) lung cancer; (**D**) other cancers; E&F. Summary of total cases. *AI* artificial intelligence, *ROC* receiver operating characteristic, *AUC* area under the curve, *PPV* positive predictive value, *NPV* negative predictive value.

As shown in Fig. 2, in subgroups divided by cancer types, our AI model displayed considerable high accuracy measured by AUC value, which was 0.955 for prostate cancer, 0.988 for breast cancer, 0.957 for lung cancer, and 0.971 for the other cancers. The age-based analysis indicated no significant diagnostic differences of bone metastasis in patients with breast cancer, lung cancer, and other cancers. However, statistically different diagnostic accuracy was investigated in patients between ≥ 60 years old (AUC = 0.938) and < 60 years old (AUC = 0.992) in prostate cancer group (*P* < 0.05). A probable reason might be the older ages of patients (71.0 ± 8.1 years) than other groups (*P* < 0.01), thus the increased risk of benign diseases in aging patients, such as osteophyte, arthrosis, osteoporotic fracture, and postoperative change, also displayed hot spots in BS and thus decreased the diagnostic accuracy of bone metastases. In addition, except for sexuality-related breast cancer and prostate cancer, there were no significant differences in the diagnosis of bone metastasis between male and female patients in lung cancer and other cancer groups.

There are still 81 misdiagnosed cases were found in the testing cohort of 1223 cases (6.62%), including 38 false-negative (3.11%) and 43 false-positive (3.51%) cases (Supplementary Table S3). Lesion number, size, and adjacent diffused signal were the major influence factors in false-negative cases. On the other hand, fracture, inflammation, degenerative, and postoperative change were the main reasons for the false-positive cases in our test.

### Human vs. AI

**Figure 3:**
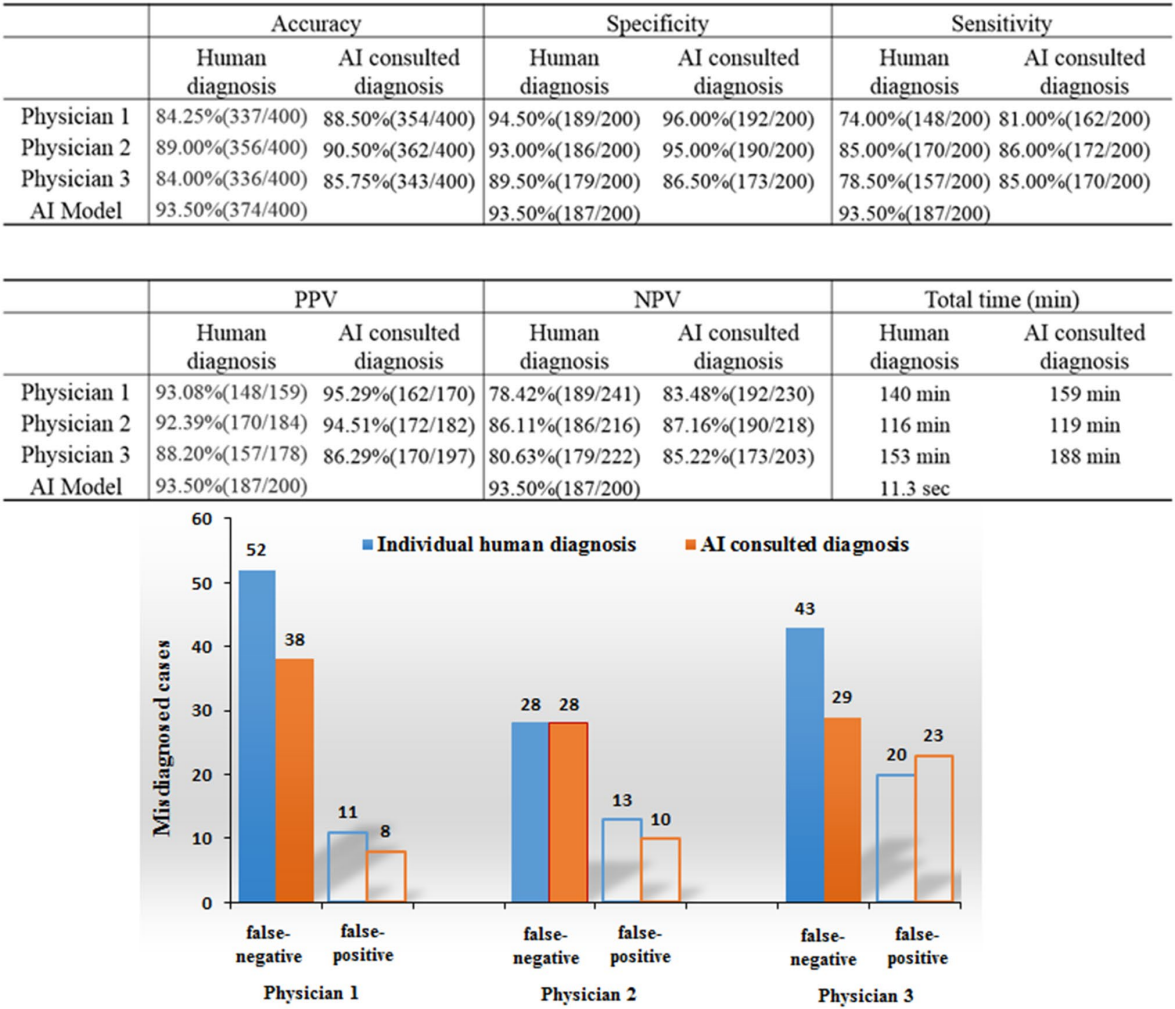
Diagnostic performance of individual diagnosis by three human physicians and human-AI consulted interpretation of 400 cases.

The comparison of diagnostic performance of AI and human physicians were shown in Fig. 3. In the interpreting competition between AI model and three qualified nuclear medicine physicians, AI model cost only 11.3 s to complete the interpretation of 400 cases, while three physicians spent 116, 140, and 153 min, respectively, to accomplish the same work, which is corresponding to a time savings of 99.88%. Then, compared with the highest performance of three physicians, AI model manifested improved accuracy (93.5% vs. 89.00%) and sensitivity (93.5% vs. 85.00%) in calculating metastases in total cases (*P* < 0.001), but the specificity between AI model (93.50%) and human (94.50%) were not significantly different. However, after consulting the AI result, physician-1 and physician-3 indicated improved diagnostic performance, especially in finding the missed lesions and reducing the false-negative rate.

In detailed error analysis, we collected 13 cases with correct interpretation by AI but misdiagnosed by all three physicians. Among these cases, 11 patients were found to have small lesions (diameter for a few millimeters) or insufficient resolution of radioactive uptake, were ignored or judged as benign by humans (Supplementary Fig. S1). The other 2 patients who had osteoporotic vertebral compression fracture were misdiagnosed as metastases by humans (Supplementary Fig. S2). Interestingly, there were 6 cases misdiagnosed by AI but correctly interpreted by all three physicians. One patient with diffuse skeletal metastasis and two patients with humerus metastases were misdiagnosed as benign by AI (Supplementary Fig. S3). Then, one patient with multiple fractures and one patient with postoperative bone change, were misdiagnosed as malignant lesions by AI model (Supplementary Fig. S4); while the last misdiagnosed case was caused by the catheter on the patient.

## Discussion

Despite the advent of various imaging modalities, such as PET/CT and multiparameter MRI, have been developed to detect skeletal metastasis, bone scintigraphies with ^99m^Tc-MDP remains one of the most effective diagnostic techniques for its considerable sensitivity and cost performance^21,22^. Skeletal imaging occupies 61.3% of 2.09 million of SPECT scans annually in China, and most of them were not fused with CT by the limited device utilization^23^. Thus, the diagnosis of BS planar image is still a challenge for the nuclear medicine physicians in China. Fortunately, an automated system might be an effective tool to overcome this dilemma. In this study, we constructed an AI model with deep neural network based on 12,222 cases to extract image features, and evaluated its efficiency for diagnosing cancer bone metastasis with BS images. This model simultaneously improved diagnostic performance and time–cost for interpreting images, and the AI consulting system could potentially improve physicians’ diagnostic skills specially for younger physicians who lacked experience. Besides, by the first time, lung cancer was separated as an individual subgroup for AI analysis and indicated diagnostic accuracy of 93.36%, which seems promising for clinical use in the future study.

Generally, deep neural networks with sufficient valid dataset is usually conducive for improving the final outcomes for AI analysis^24^. In this study, a dataset contained 12,222 BS examinations from 40 cancer types, which is the largest dataset for single-center BS image interpreting by now, was used to construct the DNN for AI modeling. Compared with traditional methods using hand-crafted features, the use of multi-input deep convolutional neural network allows AI model to follow the natural distribution, reduced subjective judgment of physicians, better generalization performance, and closer to the usual clinical environment. For example, previous studies^15,25^ usually excluded cases that could be misleading during the training process, such as patients with large bladder, sternotomy, or fracture. However, there were not any atypical cases were excluded in our dataset to help the AI model come closest to a real index. Thus, as expected, our AI model represented improved diagnostic accuracy of AUC values (0.964) compared with other BS diagnostic AI models in previous reports (0.858, 0.91, and 0.932)^18–20^. Notably, although the AI model have made false-negative of 8 cases in navigating small lesions in testing cohort, it displayed better capability in small lesion recognition than humans in following competition.

Although the AI model was able to efficiently improve the detection of missed small metastatic lesions by human and beneficial to reduce the readers’ error rates of BS interpretation, there are several limitations should be noted. First, the estimations by our AI model were based on BS images only. The false-negative and false-positive cases have still appeared, which may be due to small lesion number, lesion size, lesion adjacent to physiological uptakes like bladder, and diffused skeletal metastasis manifested by diffused homogenous uptakes. These kinds of cases were also tricky for nuclear medicine physicians to interpret based on BS images only. However, in “real” clinical works, the patients’ medical records, such as injury history, surgical record, characteristics of other imaging modalities, and the results of laboratory tests, must be considered to obtain accurate BS interpretation. According to this, the construction of a new AI model based on the fused SPECT/CT bone images is currently undergoing by our team, and we hope the addition of fused reference CT and medical records would effectively reduce the diagnostic errors. Secondly, the unsatisfied capability in recognizing add-ons on patients, such as a catheter, is still a noticeable disadvantage of this AI model but easy for physicians. Thirdly, our study just focused on the performance of the AI model on the diagnosis of absence or presence of bone metastasis to assist nuclear medicine physicians' interpretation. However, a series previous study demonstrated that the bone scan index (BSI) calculated by artificial neural networks is an effective biomarker for predicting the prognosis or survival of some malignant cancers^26–29^. Whether our AI model could be beneficial to the assessment of the prognosis or survival of some malignant cancers like BSI, it might require more concentration on lesion-based analysis. Last but not least, the retrospectively acquired database was collected from only one hospital for the present work. The patients at our hospital might not be considered typical of other centers, and the findings might be considered to be relatively institution-specific. A prospective multi-center study will also be needed to evaluate whether the AI model would be able to show satisfactory performance on BS images acquired with different gamma cameras, protocols, interpretive styles, and incidence of metastatic disease. These processes require considerable time for collecting more clinical data and will be studied in future works.

## Conclusions

Our AI model achieved considerable time-efficiency, accuracy, specificity and sensitivity in diagnosis of bone metastasis in patients with lung cancer, prostate cancer, breast cancer, and other cancers. With further assessment and validation, this model could facilitate diagnosing programs and help physicians improve the diagnostic efficiency and accuracy of bone metastasis, particularly in remote or low-resource areas, leading to a beneficial clinical impact.

## Supplementary information

Supplementary Information.

## Data Availability

Data confirming the results of this study are presented in the manuscript and are available from the corresponding author upon reasonable request.
