# Supplementary material for: Deep neural network based artificial intelligence assisted diagnosis of bone scintigraphy for cancer bone metastasis
Source: Sci Rep. 2020 Oct 12;10:17046. doi: 10.1038/s41598-020-74135-4 (PMC7550561; doi:10.1038/s41598-020-74135-4)
Supplement: Supplementary file 1 — Supplementary Information. [file 41598_2020_74135_MOESM1_ESM.docx]

**Deep Neural Network Based Artificial Intelligence Assisted Diagnosis of Bone Scintigraphy for Cancer Bone Metastasis**

Zhen Zhao^1§^, Yong Pi^2§^, Lisha Jiang^1^, Yongzhao Xiang^1^, Jianan Wei^2^, Pei Yang^1^, Wenjie Zhang^1^, Xiao Zhong^1^, Ke Zhou^1^, Yuhao Li^1^, Lin Li^1^, Yi Zhang^2^*, Huawei Cai^1^*

1. Laboratory of Clinical Nuclear Medicine, Department of Nuclear Medicine, West China Hospital of Sichuan University, Chengdu, 610041, P.R.China.
2. Machine Intelligence Laboratory, College of Computer Science, Sichuan University, Chengdu 610065, P. R. China.


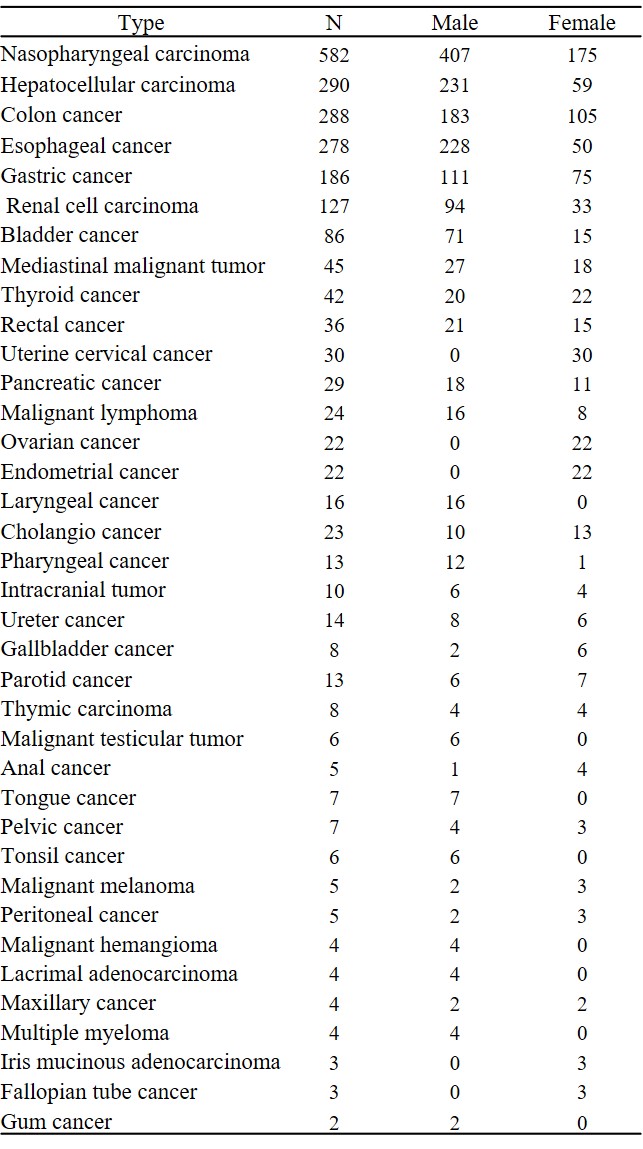


**Supplementary Table S1. Distribution of cancer type and gender in subgroup of other cancers.**

**
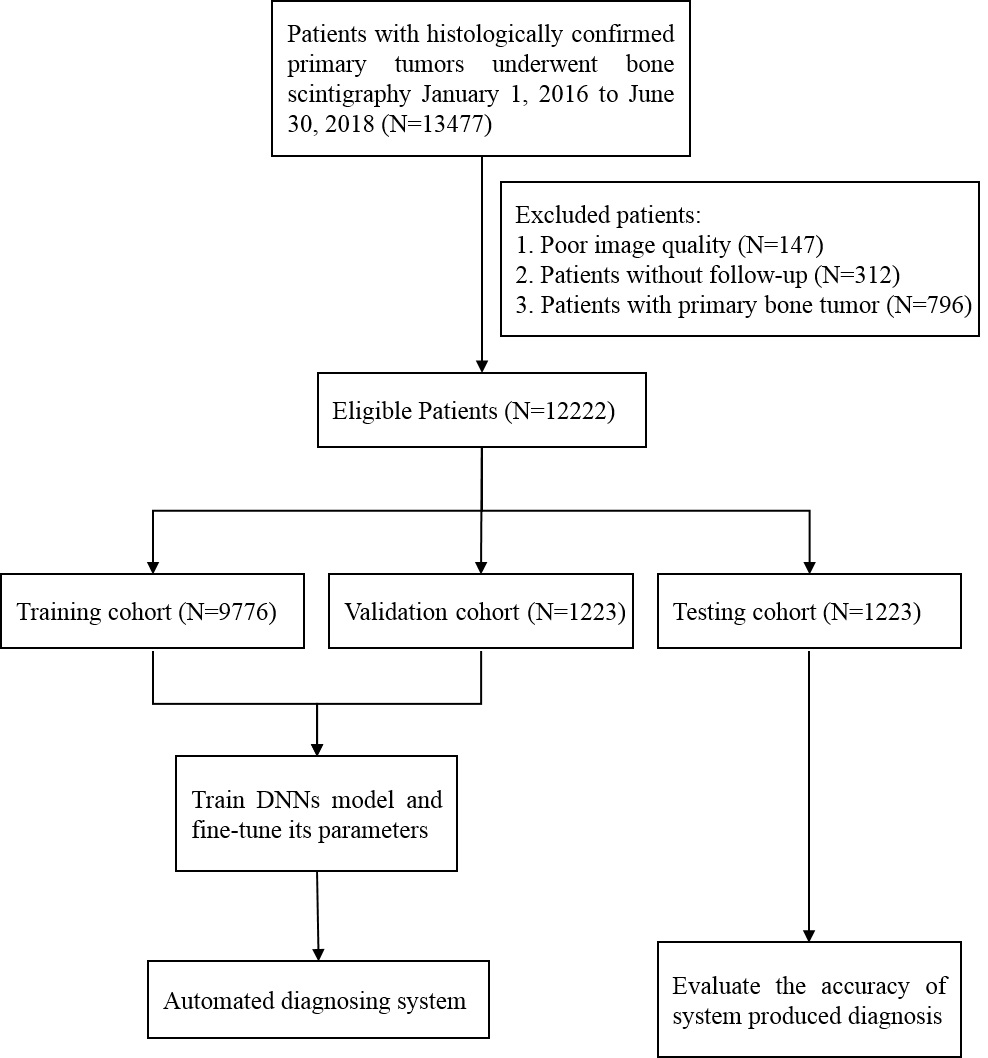
**

**Supplementary Table S2.** Diagrams of the participant flow in the study. DNNs, deep neural networks.

**
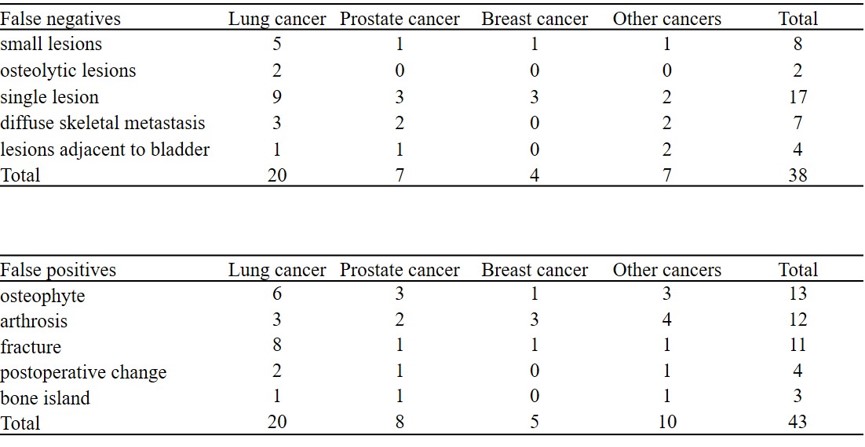
**

**Supplementary Table S3** Analysis of misdiagnosed cases by AI model. Seventeen patients displayed only one single lesion and 8 patients with small lesion (diameter ≤5 mm) were ignored by AI model. Then, 4 patients had the lesions adjacent to the urinary bladder was judged as diffused urinary bladder uptake by the model, and the other 7 patients developed diffuse skeletal metastasis were misdiagnosed as benign. Besides, 2 patients who did not have radionuclide accumulation in bones because of osteolytic lesions with little osteoblastic component were ignored as well. While the false-positive cases included 13 patients with osteophyte, 12 patients with arthrosis, 11 patients with fracture, 4 patients with postoperative change and 3 patients with bone islands.


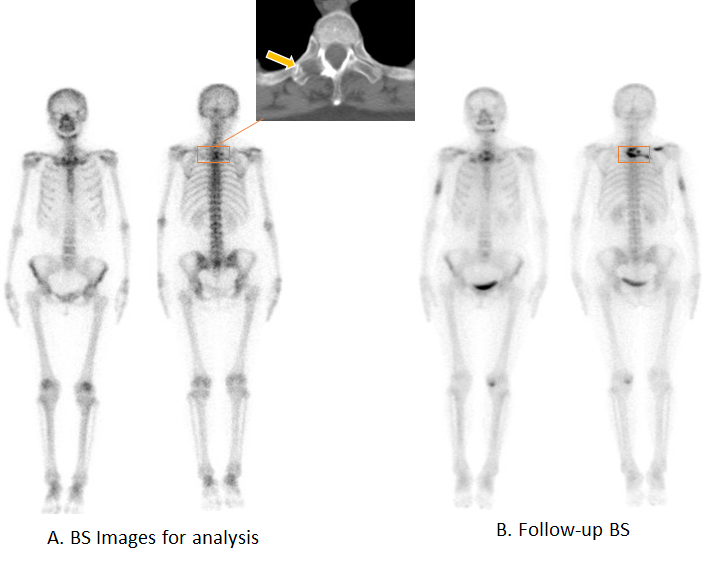


**Supplementary Fig. S1.** Representative false-negative case by human but correct by AI model. A 59-year-old woman with lung cancer was diagnosed with bone scintigraphy for evaluating possible bone metastasis. Whole-body bone planar scintigraphy (A) after injection of ^99m^Tc-MDP revealed a little focal uptake in T3 thoracic vertebra. The lesions was diagnosed as benign by all three experts but corrected as malignant by AI model. CT image displayed the destruction of transverse process and pedicle in T3. Eight months later, follow-up bone scintigraphy (B) was obtained, and disclosed intensely distributions of ^99m^Tc signals were found in the thoracic vertebra and new lesions in right clavicle and humerus. The final diagnosis was bone metastasis.

**
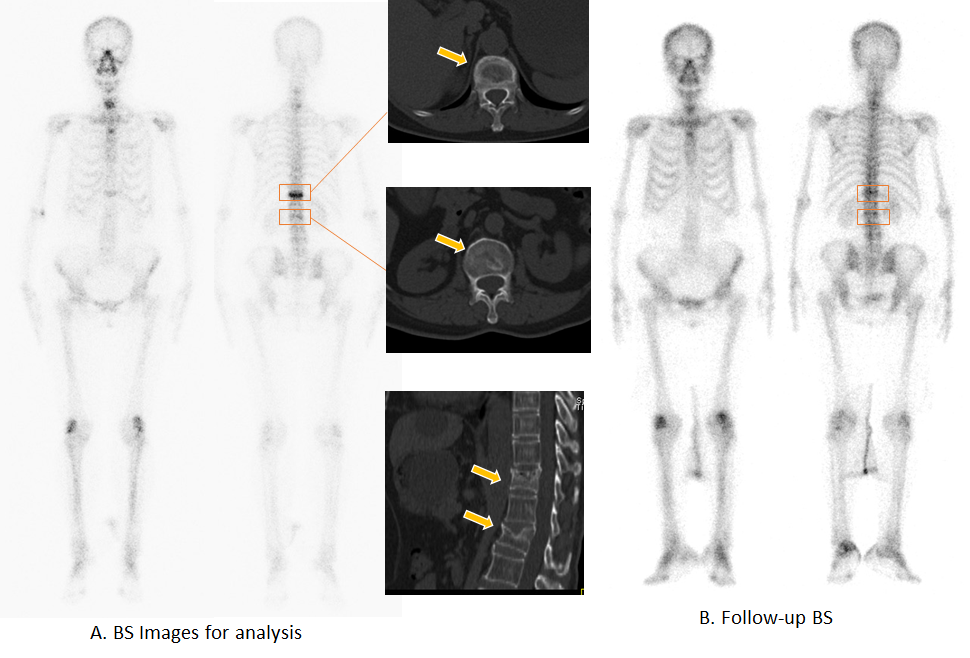
**

**Supplementary Fig. S2.** Representative false-positive case by human but correct by AI model. A 69-year-old woman with left-sided breast cancer after surgery was diagnosed with bone scintigraphy for evaluating possible bone metastasis. Whole-body bone planar scintigraphy after injection of ^99m^Tc-MDP revealed intense uptake in T12 and L2. The lesions were diagnosed as malignant by three experts but judged as benign by AI. CT image displayed the cortex of T12 and L2 was discontinuous. Sixteen months later, follow-up bone scintigraphy (B) revealed declined uptake at original lesion. The final diagnosis was compressive fracture.


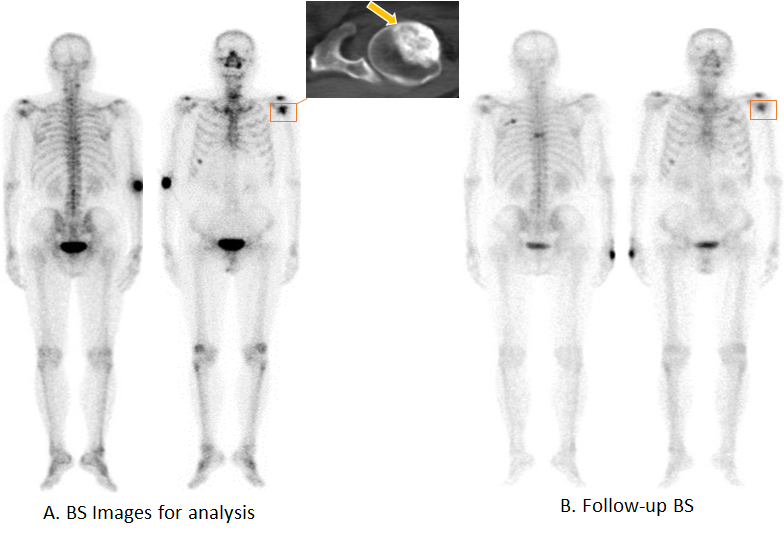


**Supplementary Fig. S3.** Representative false-negative case by AI model but correct by human. A 68-year-old man with prostate cancer was diagnosed with bone scintigraphy for evaluating possible bone metastasis. Whole-body planar bone scintigraphy (A) showed intense uptake in the upper part of left humerus. The lesion was diagnosed as benign by AI model but malignant by all three experts. Further CT images revealed osteogenic bone destruction in left humerus. Six months later, follow-up bone scintigraphy (B) disclosed persistent uptake in left humerus and new lesions in left rib and thoracic spine. The final diagnosis was bone metastasis.

**
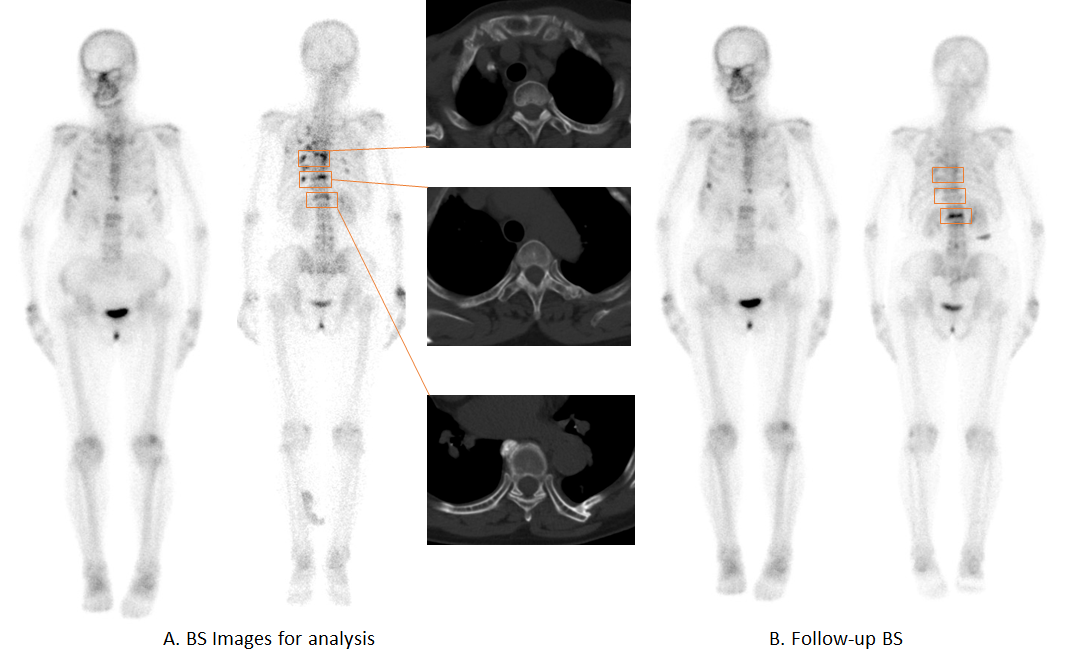
**

**Supplementary** **Fig. S4.** Representative false-positive case by AI correct by human. A 64-year old woman with right-sided breast cancer was diagnosed with bone scintigraphy for evaluating bone metastasis after surgery. Whole-body bone scintigraphy (A) revealed intensely focal uptake in multiple ribs and thoracic vertebrae. Subsequent CT results displayed multiple fractures in ribs and thoracic vertebrae. Seven months later, follow-up bone scintigraphy (B) indicated disclosed slight uptake in all rib lesions and most of thoracic vertebrae lesions. The final diagnosis was multiple fractures.
